# G‑Quadruplex and i‑Motif Structures in the *SHMT1* 5′UTR Modulate Gene Expression

**DOI:** 10.1021/acsomega.5c13146

**Published:** 2026-03-19

**Authors:** Rosalia M. Palumbo, Manju Kasaju, Sophia C. Hershey, Morgan E. McCann, Zoe H. Woon, David B. Heisler, Mihaela-Rita Mihailescu

**Affiliations:** † Department of Chemistry & Biochemistry, 6613 Duquesne University, Pittsburgh, Pennsylvania 15282, United States; ‡ Department of Chemistry, Bryn Mawr College, Bryn Mawr, Pennsylvania 19010, United States

## Abstract

Multiple sclerosis is a fatal neurodegenerative disease that progresses by eroding the myelin sheath and exposing the neuron, leading to neuronal degradation and death. While multiple sclerosis remains without an effective treatment or cure, studies have identified genes that are dysregulated in multiple sclerosis patients and predicted to be involved with disease progression. These genes are primarily involved in controlling DNA methylation, a process required for regulating gene expression that is critical for cellular health. Having identified potential genetic risk factors, current research focuses on how to manipulate the expression of these genes, offsetting DNA methylation errors in patients by targeting DNA secondary structure formation. Serine hydroxymethyltransferase 1 (SHMT1) is a key player in DNA methylation and was determined to be upregulated in multiple sclerosis patients. Here, we characterized hybrid 3 + 1 G-quadruplex (GQ) and i-motif (iM) structures in the *SHMT1* DNA 5′ untranslated region and a parallel GQ in the corresponding mRNA. Additionally, we found that the GQ/iM structures suppress the mRNA levels and protein expression of a reporter gene. Together, these data suggest that GQ/iM structures are necessary for *SHMT1* regulation, which could serve as a target for therapeutic intervention for multiple sclerosis patients.

## Graphical abstract

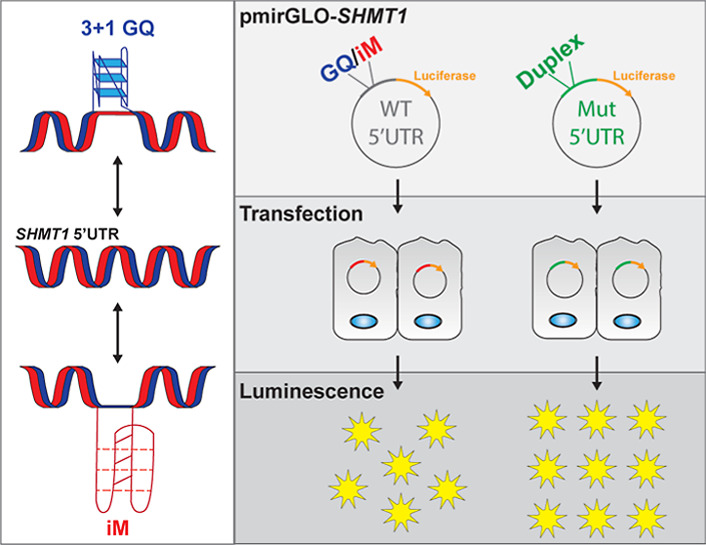

## Introduction

Multiple sclerosis (MS) is a chronic autoimmune and neurodegenerative disease of the central nervous system that directly targets and destroys the protective myelin sheath. MS, which is currently incurable, affects ∼2.8 million people worldwide,^1^ with patients typically being diagnosed between the ages of 20 and 50 years, three-quarters of whom are women.^2^ Symptoms include vision loss due to optic neuritis, ataxia, paresthesia, pain, and fatigue, with roughly half of MS patients also experiencing cognitive impairment.^3^ The exact cause of MS remains unknown, but a combination of genetic and/or environmental factors (low vitamin D, obesity, smoking, viral infection, etc.) are suspected to contribute.^4^ Since only about a quarter of MS patients have known MS genetic risk loci, dysregulation at the epigenetic level through DNA methylation, histones, and other proteins may play an important role in the etiology of the disease.^5,6^

DNA methylation, or the methylation of cytosines located at CpG sites to 5-methyl-cytosine (5-mC), typically represses gene expression. The exact mechanism by which DNA methylation silences gene expression is not fully understood, but two possibilities have been proposed: (i) formation of 5-mC prevents the binding of transcription factors to their target sites^5^ or (ii) DNA-binding proteins containing methylated DNA-binding domains bind to 5-mC and recruit transcriptional silencing complexes.^7^ Genome-wide association studies (GWAS) comparing gene expression in patients with different types of MS to healthy patient controls have identified changes in DNA methylation patterns as a common factor.^8,9^ Additionally, studies have identified that proteins involved in regulating DNA methylation are abnormally expressed in MS patients, including serine hydroxymethyltransferase 1 (SHMT1), solute carrier family 19 member 1 (SLC19A1),^10^ DNA methyltransferases (DNMTs),^11,12^ ten-eleven translocases 1 to 3 (TET1-3),^12,13^ and methylated DNA binding domain 2 protein (MBD2).^13^ In this study, we focused on SHMT1, since the *SHMT1* rs4925166 single nucleotide polymorphism has been identified as a susceptibility locus for MS, and a subsequent study determined the upregulation of SHMT1 in white matter lesions of MS patients.^14,15^

SHMT1 contributes to DNA methylation via participation in the folate cycle^16^ by regulating the availability of the methyl donor *S*-adenosylmethionine (SAM). SHMT1 catalyzes the conversion of tetrahydrofolate (THF) to 5,10-methylene THF (5,10-mTHF) via the addition of a single carbon (Figure 1).^7,17^ 5,10-mTHF is then processed into 5-methyl THF (5-mTHF), the methyl donor for methionine formation from homocysteine.^7^ Methionine is critical for DNA methylation, as it is the precursor to SAM, one of the most prolific methyl donors in the cell. The increased expression of SHMT1 in MS patients could indicate a possible correlation between the expression of this gene and the abnormal DNA methylation status.^14,15^ Therefore, understanding the regulatory mechanisms of *SHMT1* expression could lead to a potential new target for MS therapeutic intervention.

**Figure 1:**
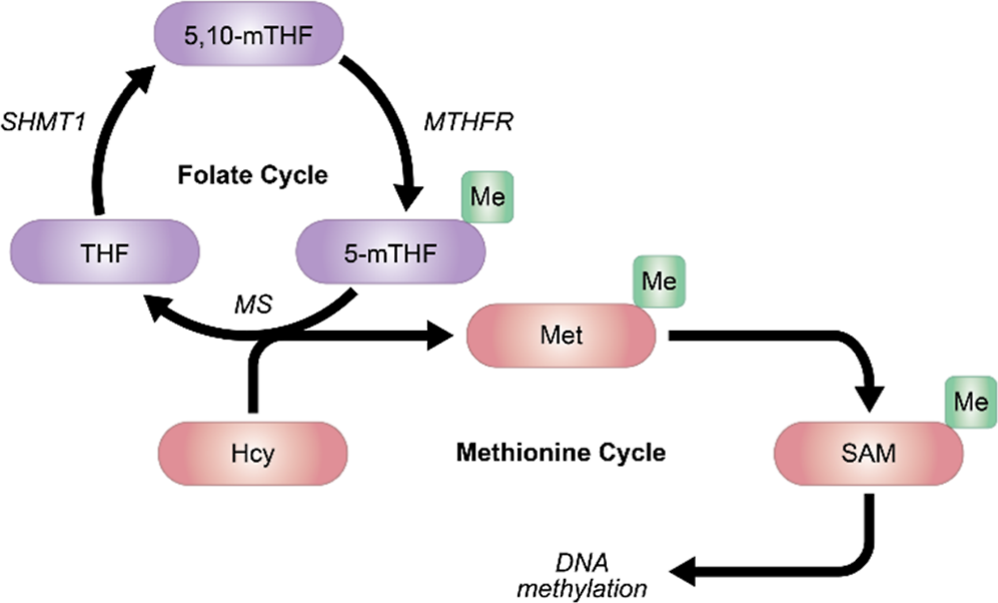
Role of SHMT1 in the DNA methylation cycle. SHMT1 catalyzes the conversion of tetrahydrofolate (THF) to 5,10-methylene THF (5,10-mTHF), which is then methylated to become 5-methyl THF (5-mTHF) by methylenetetrahydrofolate reductase (*MTHFR*). 5-mTHF enters the methionine cycle as a precursor to *S*-adenosylmethionine (SAM), a prolific methyl donor necessary for DNA methylation in the cell. 5-mTHF is converted back into THF by methionine synthase (*MS*) to complete the folate cycle. In the methionine cycle, production of SAM is achieved via the methylation of homocysteine (Hcy) to methionine (Met), which is then converted into the final methyl donor SAM.

An emerging route for new treatments is transcriptional and translational control of gene expression via noncanonical DNA/RNA secondary structures, such as guanine-quadruplexes (GQs) and intercalated-motifs (iMs). GQs and iMs are rich in guanines or cytosines, respectively, and rely on noncanonical hydrogen bonding base pair formation. GQs are formed within DNA or RNA sequences with at least four G-tracts (G_≥3_N_*x*_G_≥3_N_*x*_G_≥3_N_*x*_G_≥3_) that are separated by loops of variable nucleotide length (*x* = 1 to 12). Four Gs engage in Hoogsteen base pairing, forming G-tetrads that stack upon each other and are stabilized by central potassium ions.^18,19^ GQs are prevalent in the human genome, with over 700,000 unique GQs (with loops of 1–12 nucleotides) predicted to form with high density in promoters, 5′ untranslated regions (5′UTRs), and splicing sites.^20,21^ iMs are formed by the hemiprotonation of cytosine bases, which allows two cytosines to hydrogen bond and intercalate upon another C–C base pair.^22,23^ Hemiprotonation occurs only at low pH (4.0–6.0), causing iM formation *in vivo* to be debated. However, a recent study identified over 53,000 iM structures at the same genomic sites across three different human cell lines,^24^ likely due to molecular crowding and/or the presence of magnesium ions.^25,26^ While it has been shown that steric hindrance prevents GQs and iMs from forming simultaneously directly across from each other, sequences that have more than four G-rich and C-rich repeats can form simultaneously in a staggered fashion.^27^

It is well-established that these noncanonical nucleic acid secondary structures regulate gene expression both at the transcriptional and translational levels. In general, DNA GQs have been found to inhibit transcription, while iMs activate it, but this trend is not universal. In some cases, GQs are suggested to form “binding hubs” for transcription factors that upregulate gene expression,^28^ and some iMs have been found to repress transcription.^29,30^ Thus, in a gene-dependent manner, DNA GQ and iM structures could function as molecular switches for controlling transcriptional regulation. Moreover, GQs formed in RNA have been found to regulate mRNA translation, alternative splicing, 3′-end processing, and alternative polyadenylation.^31–34^

Here, we analyzed *SHMT1* for sequences that could form GQ/iMs and identified a region within its 5′UTR that exhibited strong potential for DNA GQ/iM and RNA GQ formation. Given this, we hypothesized that DNA GQ/iM and RNA GQ structures form in the 5′UTR of *SHMT1* and regulate its expression. We used biophysical methods to show that the G-rich sequence in the *SHMT1* DNA 5′UTR forms a stable hybrid 3 + 1 GQ exhibiting potassium-dependent stability. The complementary *SHMT1* DNA C-rich sequence forms an iM structure that is highly stable at pH values of 4.0 and 5.5. Additionally, we found that the corresponding G-rich mRNA sequence forms a parallel GQ stabilized in the presence of potassium. Moreover, through luciferase reporter assays, we demonstrate that these noncanonical structures suppress gene expression, potentially acting as a molecular switch that could be targeted for therapeutic intervention against MS.

## Results and Discussion

### The *SHMT1* 5′UTR DNA G-Rich Sequence Forms a Stable Hybrid 3 + 1 G-Quadruplex Structure in the Presence of Potassium Ions

Given that MS patients exhibit altered DNA methylation, we analyzed genes involved in the methylation pathway for regions that could fold into GQ structures and identified a 25-nucleotide-long G-rich region in the 5′UTR of the *SHMT1* gene. When assessed with the GQ-predictive QGRS Mapper software, this sequence was predicted to form a GQ structure (G-score: 40).^35^ The G-score rewards a larger number of Gs within a repeat and shorter intervening loops.^35^ For reference, the highest possible predicted G-score by this software for a 30-nucleotide sequence is 105, corresponding to a GQ with six G quartets, and we previously characterized three-plane GQs with predicted G-scores of ∼40 ^36^ and four-plane GQs with predicted G-scores of ∼55.^37^ Therefore, given its high predicted propensity to fold into a GQ, the *SHMT1* DNA G-rich sequence (named here *SHMT1* DNA GR, Table 1) was characterized by various biophysical methods.

**Table 1:** *SHMT1* DNA, *SHMT1* DNA Mutant, and *SHMT1* mRNA Sequences as well as GQ-Positive and -Negative Controls Used in This Study^a^

Gene name	Structure	Sequence
*SHMT1* DNA GR	GQ	5′-**GGGG**CGTT**GGG** TCAGC **GGG**TCT**GGG**-3′
*SHMT1* DNA CR	iM	3′-**CCCC**GCAA**CCC** AGTCG **CCC**AGA**CCC**-5′
*SHMT1* GR_MUT	GQ mutant	5′-**GACG**CGTT**GAT** TCAGC **CTG**TCT**GAC**-3′
*SHMT1* CR_MUT	iM mutant	3′-**CTGC**GCAA**CTA** AGTCG **GAC**AGA**CTG**-5′
*SHMT1* RNA GR	GQ	5′-**GGGG**CGUU**GGG**UCAGC**GGG**UCU**GGG**-3′
*BDNF* RNA	GQ+ control	5′-**GGG**AU**GGGGG**AU**GGGGGG**-3′
*NEAT1* RNA	GQ+ control	5′-**GGG**A**GGG**A**GGG**A**GGG**AGGCGG-3′
*SARS-CoV-2* open reading frame 1a	GQ– control	5′-GACUGUAGUGCGCGUC-3′
*SARS-CoV-2* s2m	GQ– control	5′-UCACCGAGGCCACGCGGAGUACGAUCGAGUGUACAG UGAA-3′

a The Gs highlighted in bold are predicted to form GQs, and the Cs highlighted in bold are predicted to form iMs. The underlined nucleotides were mutated to disrupt GQ/iM formation.

We first used one-dimensional (1D) proton nuclear magnetic resonance (^1^H NMR) spectroscopy to determine if *SHMT1* DNA GR formed a GQ by monitoring the imino proton resonance region between 10 and 12 parts per million (ppm), which corresponds to G imino protons involved in Hoogsteen base pairs within the G-tetrads.^36,38^ We observed multiple imino proton resonances in the 10–12 ppm range, even in the absence of potassium ions, indicative of the formation of the GQ structure (Figure 2A). Upon the addition of 10 mM KCl, the appearance of a new set of resonances was observed, and with each subsequent addition of KCl, the new resonances increased in intensity with a concomitant decrease in intensity of some of the resonances observed at 0 mM KCl. These results suggest that the addition of potassium ions promotes the formation of a different GQ structure, unique from the one formed in the absence of potassium.

**Figure 2:**
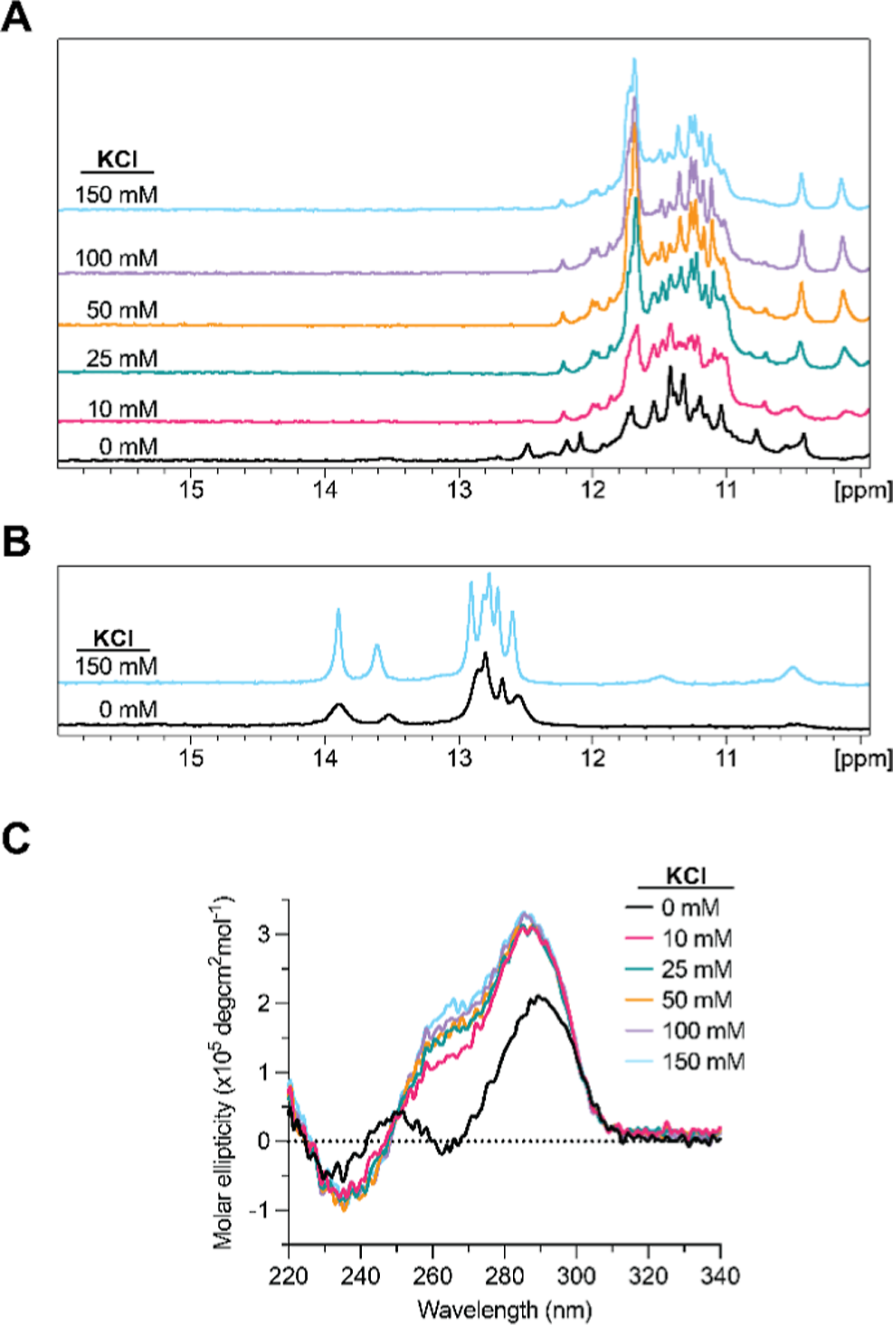
(A) KCl dependence of 1D ^1^H NMR spectroscopy of the *SHMT1* DNA GR sequence. In the absence of KCl, resonances in the 10–12 ppm range are indicative of GQ formation. Significant resonance changes occur upon the addition of 10 mM KCl, and the intensity of these resonances increases with increasing KCl concentration, suggesting a change to the GQ structure further stabilized by KCl. (B) KCl dependence of 1D ^1^H NMR spectroscopy of the *SHMT1* GR_MUT sequence. Mutation of critical nucleotides in the G-tracts results in loss of the GQ resonances in the 10–12 ppm region and appearance of resonances corresponding to Watson–Crick base pairing (12–14.5 ppm). (C) KCl dependence of CD spectroscopy results of the *SHMT1* DNA GR sequence. In the absence of KCl, a characteristic antiparallel GQ signature is observed (∼295 nm max, ∼260 nm min). Upon addition of KCl, the signature shifts to that of a stable 3 + 1 hybrid GQ structure (∼295 and ∼265 nm max, ∼240 nm min), which persists to 150 mM KCl.

To confirm that the predicted G-repeats are responsible for the GQ structure, we designed a control mutant *SHMT1* GR sequence which had several Gs mutated (*SHMT1* GR_MUT, Table 1). As expected, analysis by ^1^H NMR spectroscopy revealed that the broad imino proton resonances present in *SHMT1* DNA GR in the 10–12 ppm range are absent from the spectra of *SHMT1* GR_MUT at both 0 and 150 mM KCl (Figure 2B). In contrast, imino proton resonances in the 12–14.5 ppm region, corresponding to Watson–Crick base pairing, are present at 0 mM KCl and become sharper at 150 mM KCl, indicating hairpin or duplex structure formation. We analyzed potential unimolecular and bimolecular folds of this sequence using RNAstructure software, which suggested stable intramolecular hairpin and intermolecular duplex structures which could give rise to these resonances in the NMR spectra (Figure S1). These results demonstrate that the mutated nucleotides disrupt the G-tracts of this sequence and prevent the formation of the GQ structure even at 150 mM KCl.

Next, we used circular dichroism (CD) spectroscopy to characterize the orientation of the GQ structure, as CD spectra can differentiate between the three unique GQ conformations (parallel, antiparallel, and hybrid 3 + 1).^39,40^ A parallel GQ structure exhibits a positive maximum at 265 nm and a negative minimum at 240 nm, while an antiparallel formation results in a positive maximum at 295 nm and a negative minimum at 260 nm.^41^ A hybrid 3 + 1 GQ conformation gives rise to positive maxima at ∼295 nm and ∼260 nm with a negative minimum at ∼245 nm.^41^ The spectrum of *SHMT1* DNA GR in the absence of potassium exhibited the characteristic signature of an antiparallel GQ, with a positive maximum at ∼295 nm and a negative minimum at ∼260 nm (Figure 2C). However, upon the addition of KCl, the spectrum shifted significantly, resulting in positive maxima at ∼295 and 265 nm with a negative minimum at ∼240 nm. Further additions of KCl caused a slight increase in the intensity of the positive bands at 295 and 265 nm. These results correlate with a GQ structure that shifts from an antiparallel orientation to a stable hybrid 3 + 1 orientation in the presence of potassium ions. This finding is consistent with our ^1^H NMR results, which indicated a conformational change to the GQ structure that is further stabilized by increasing KCl concentrations.

Next, we analyzed the *SHMT1* DNA GR GQ by native polyacrylamide gel electrophoresis (PAGE) in the presence of increasing KCl concentrations. When the gel was stained with *N*-methylmesoporphyrin IX (NMM), which has been shown to only effectively stain parallel GQs,^42,43^ no visible bands were observed in the lanes containing *SHMT1* DNA GR samples even though a GQ-positive control was visible (Figure S2). Although *SHMT1* DNA GR forms two distinct and stable GQ conformations, neither is stained by NMM, which supports the formation of an antiparallel GQ that shifts into a hybrid 3 + 1 conformation in the presence of potassium ions.

Finally, we used ultraviolet (UV) thermal denaturation spectroscopy to evaluate the effect of potassium ions on the stability of *SHMT1* DNA GR by monitoring the change in absorbance at 295 nm with increasing temperature. At all KCl concentrations investigated, the *SHMT1* DNA GR denaturation gave rise to a hypochromic transition, the signature of GQ dissociation (Figure 3A).^44^ In the absence of KCl, the melting temperature, *T*_m_, was determined to be ∼35 °C (eqs 1 and 2, Materials and Methods), increasing to 56 °C after the addition of 10 mM KCl (Table S1). This result suggests that the hybrid 3 + 1 GQ formed by *SHMT1* DNA GR in the presence of KCl is significantly more stable than the antiparallel GQ formed in the absence of potassium ions. Moreover, the *T*_m_ of the hybrid 3 + 1 GQ further increased in the presence of increasing KCl concentrations, reaching ∼71 °C at 150 mM KCl (Table S1).

**Figure 3:**
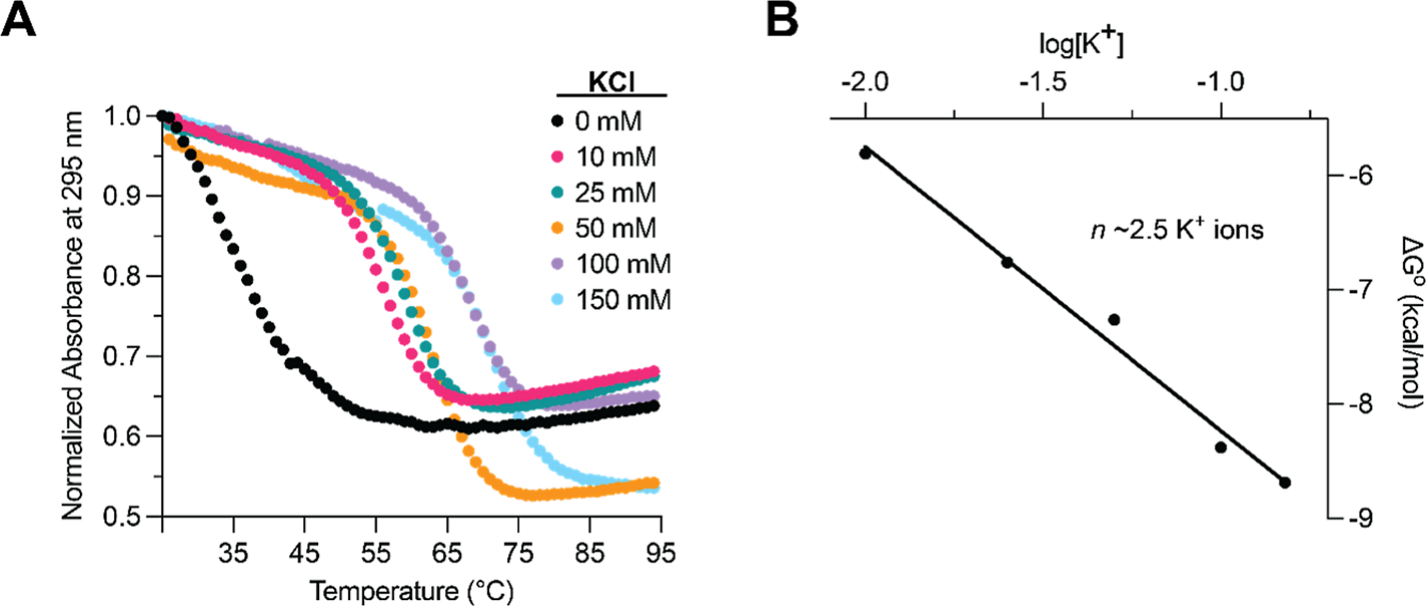
(A) KCl dependence of UV thermal denaturation of the *SHMT1* DNA GR sequence. Hypochromic transitions of GQ unfolding are observed at each KCl concentration, with each titration subsequently displaying a higher *T*_m_, indicating KCl-dependent stability. (B) Plot of Δ*G*° versus log­[K^+^] for *SHMT1* DNA GR UV thermal denaturation. The slope of the graph, *n*, is equal to the average number of potassium ions intercalated into the *SHMT1* DNA GQ structure.

The standard enthalpy, entropy, and free energy (Δ*H*°, Δ*S*°, Δ*G*°) of the *SHMT1* DNA GR folding were calculated by fitting the hypochromic transition measured in the presence of each KCl concentration to eq 1, which assumes a two-state model, and are reported in Table S1. Reported values for the enthalpy of formation of a single G-quartet plane are from −18 to −25 kcal/mol,^45^ which, compared to our results of −64.7 ± 0.1 kcal/mol for 150 mM KCl, suggest that *SHMT1* DNA GR forms a three-plane GQ structure. From the slope of the calculated free energy of the GQ structure folding, plotted as a function of the logarithm of the KCl concentration (Figure 3B), we estimate that there are ∼2.5 K^+^ ions incorporated in the *SHMT1* DNA GQ structure, which is consistent with a three-plane GQ (eq 3, Materials and Methods). In summary, we show that in the presence of KCl, *SHMT1* DNA GR forms a stable hybrid 3 + 1 GQ structure, which coordinates on average ∼2.5 K^+^ ions.

### The *SHMT1* 5′UTR DNA C-Rich Sequence Forms a Stable i-Motif Structure

The C-rich *SHMT1* sequence (named here *SHMT1* DNA CR, Table 1), complementary to the G-rich sequence forming a GQ, was analyzed for its potential to fold into an iM structure by using biophysical methods. These experiments were performed at varying pH values because iM formation relies on the hemiprotonation of one cytosine in each structural base pair.

We first used 1D ^1^H NMR spectroscopy, since hemiprotonated cytosines in iM structures give rise to unique proton resonances in the 15–16 ppm range. The ^1^H NMR spectra of *SHMT1* DNA CR at pH 4.0 and 5.5 showed resonances in the 15–16 ppm range (Figure 4A). We also observed two resonances in the 10.4–10.6 ppm range, which we attribute to G–G or T–T interactions made possible by the loops of the iM structure.^46^ At pH 6.5, the resonances in the region 15–16 ppm lose intensity, suggesting the destabilization of the iM structure, with the concomitant appearance of multiple new resonances in the region 12–14.5 ppm. This range corresponds to Watson–Crick A–T and G–C base pairs that become possible at this higher pH, potentially forming between the loops of the iM structure. However, these resonances are broad and have low intensity, suggesting that these loop–loop interactions are dynamic and transient in nature. At pH 7.0, both the iM and the Watson–Crick resonances are lost, indicating that the Watson–Crick base pairing was dependent on the presence of the iM structure scaffold and that *SHMT1* DNA CR lacks any secondary structure under these conditions.

**Figure 4:**
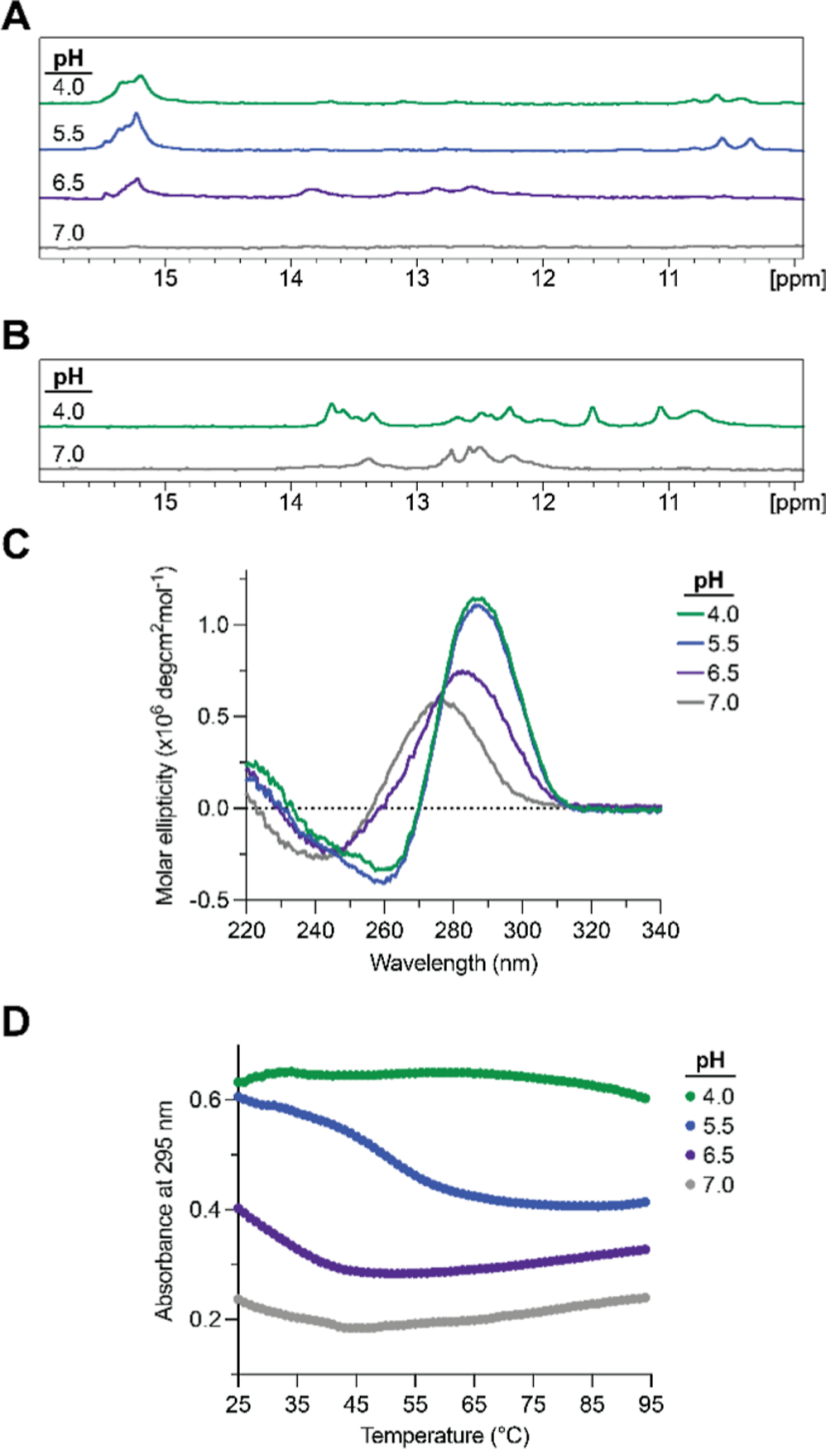
(A) pH dependence of 1D ^1^H NMR spectroscopy of the *SHMT1* DNA CR sequence. In acidic conditions (pH 4.0, 5.5), resonances are present in the 15–16 ppm range, indicative of iM formation. At pH 6.5, the iM resonances lose intensity, and Watson–Crick resonances appear (12–14 ppm). At pH 7.0, the iM resonances are lost. (B) KCl dependence of 1D ^1^H NMR spectroscopy of the *SHMT1* CR_MUT sequence. Mutation of critical nucleotides in the C-tracts results in loss of the iM resonances in the 15–16 ppm region and appearance of Watson–Crick resonances in the 12–14 ppm range. (C) pH dependence of CD spectroscopy results of the *SHMT1* DNA CR sequence. The strong iM signature (290 nm max, 260 nm min) at pH 4.0 and 5.0 is gradually lost at pH 6.5 and is fully eliminated at pH 7.0. (D) pH dependence of UV thermal denaturation results of the *SHMT1* DNA CR sequence. The hypochromic transition at pH 5.5 corresponds to the denaturation of the iM structure. The pH 4.0 iM structure was too stable to denature under these conditions.

In control experiments, we mutated *SHMT1* DNA CR to disrupt the C-tracts predicted to be involved in iM formation (*SHMT1* CR_MUT, Table 1). As expected, the iM signature resonances in the 15–16 ppm region are absent from the 1D ^1^H NMR spectrum of *SHMT1* CR_MUT at pH 4.0 (Figure 4B). Multiple resonances are present in the ∼11–14.5 ppm range at both pH 4.0 and 7.0, suggesting that this sequence forms A–T, G–C, and G–T base pairs. RNAstructure analysis of the *SHMT1* CR_MUT sequence predicted intramolecular hairpin and intermolecular duplex formation, which could account for the resonances in the Watson–Crick range (Figure S3). These results confirm that the selected nucleotide mutations within the C-tracts of this sequence effectively eliminate the formation of iM structures, even at low pH levels.

To further characterize the *SHMT1* DNA CR iM structure, we used CD spectroscopy, as iM structures have a distinct CD signature with a positive maximum at 290 nm and a negative minimum at 260 nm. At pH 4.0 and 5.5, the *SHMT1* DNA CR spectra showed a positive maximum at ∼290 nm and a negative minimum at ∼260 nm, indicating that the iM forms at low pH conditions (Figure 4C). However, at pH 6.5, the signal intensity dropped significantly, and the bands shifted to a positive maximum at ∼285 nm and a negative minimum near ∼240 nm. At pH 7.0, the iM signature is completely lost, and the bands shifted further to a positive maximum at ∼275 nm and a negative minimum at ∼240 nm. These results are consistent with the ^1^H NMR spectroscopy results, which indicated a stable iM structure at pH 4.0 and 5.5, which becomes destabilized at pH 6.5 and unfolded at pH 7.0.

We then evaluated the stability of the *SHMT1* DNA iM structure at each pH level by UV thermal denaturation spectroscopy, as iM unfolding exhibits a hypochromic transition at 295 nm. At pH 4.0, the *SHMT1* DNA CR iM structure was too stable to denature within the measured range, with minimal destabilization at the highest temperatures (Figure 4D). A full hypochromic curve was observed for *SHMT1* DNA CR at pH 5.5, which was fit to eq 1 to determine a *T*_m_ of 49.9 ± 0.1 °C and the corresponding thermodynamic parameters for iM folding (Table S2). The curves at pH 6.5 and 7.0 show the end tail of a hypochromic transition, indicating that the iM is unstable at these pH values. Taken together, these data confirm the formation of an iM structure in *SHMT1* DNA CR that is highly stable at pH 4.0 and 5.5.

### The *SHMT1* 5′UTR mRNA G-Rich Sequence Forms a Stable Parallel G-Quadruplex Structure Even in the Absence of Potassium

Given that the *SHMT1* DNA GR sequence is located on the coding strand, we investigated the possibility that the corresponding GR mRNA sequence could also form a GQ structure. Considering the emerging studies showing these secondary structures forming in mRNA 5′UTRs and acting as potential translational regulators,^47^ we implemented the same biophysical techniques to characterize this *SHMT1* RNA GR sequence (Table 1). The 1D ^1^H NMR spectra of *SHMT1* RNA GR showed GQ-signature resonances in the 10–12 ppm region, even at 0 mM KCl (Figure 5A). These resonances initially became sharper upon the first addition of KCl, indicating GQ stabilization, after which they became broader with increasing KCl concentrations. We attribute the line broadening and loss of intensity of these resonances to intermolecular GQ stacking interactions stabilized by the high concentration of potassium ions. The formation of such higher-molecular-weight complexes leads to their slower tumbling and a faster relaxation of the transverse magnetization due to enhanced spin–spin interactions and hence the broadening and reduced intensity of the signal. No Watson–Crick resonances (12–14.5 ppm) were present at any KCl concentration, indicating no competing hairpin structure is formed by *SHMT1* RNA GR.

**Figure 5:**
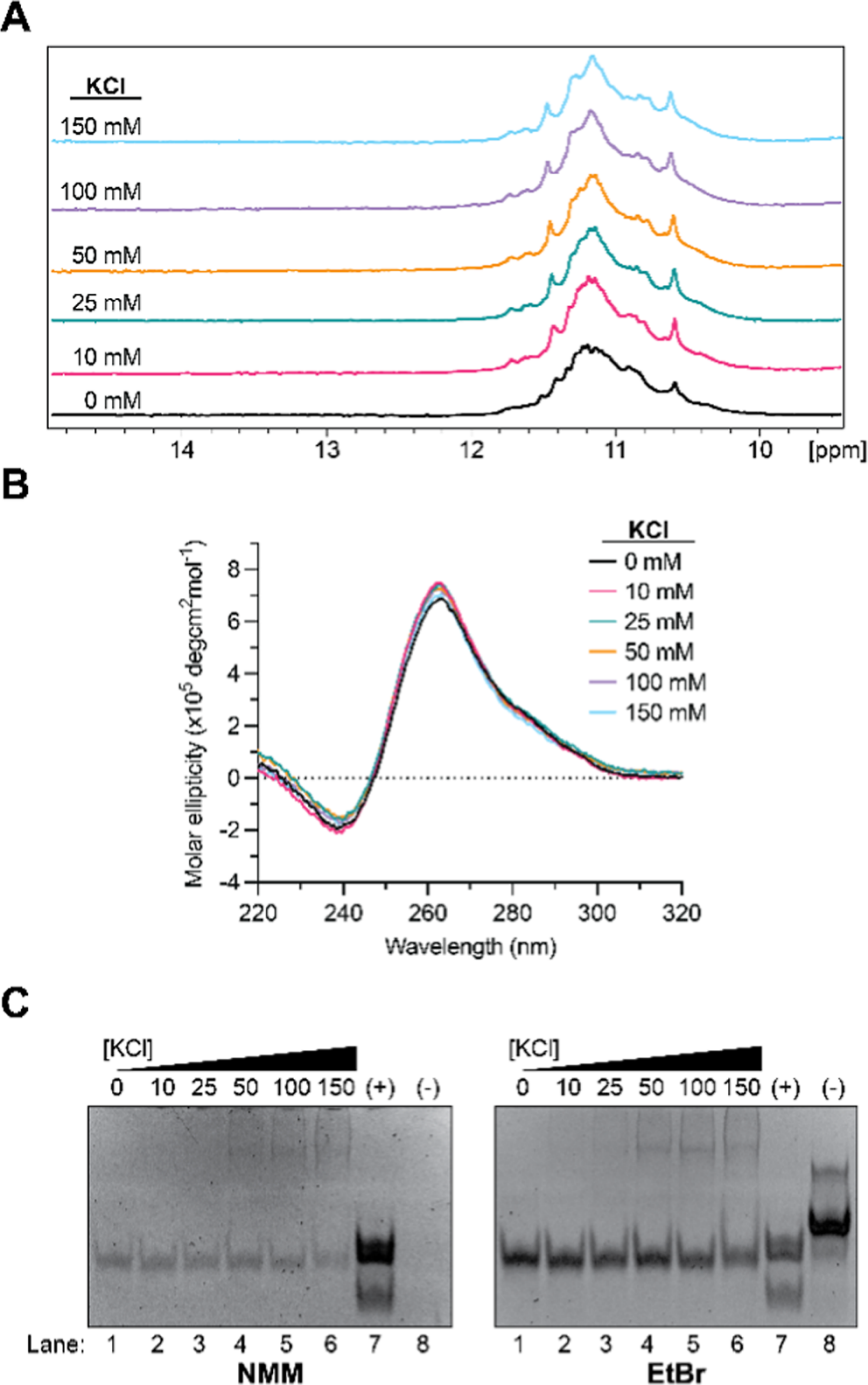
(A) KCl dependence of 1D ^1^H NMR spectroscopy of the *SHMT1* RNA GR sequence. Imino proton resonances in the 10–12 ppm region, even in the absence of potassium, indicate stable GQ formation. (B) KCl dependence of CD spectroscopy of the *SHMT1* RNA GR sequence. The RNA GQ exhibits characteristic parallel GQ signals of a ∼265 nm maximum and a 240 nm minimum under all KCl concentration conditions. (C) KCl dependence of the native PAGE of the *SHMT1* RNA GR sequence. The presence of bands in the NMM staining of *SHMT1* (lanes 1–6) compared to a positive GQ control (lane 7) confirms the GQ formation seen in the CD and NMR spectroscopy results.

Next, we used CD spectroscopy to determine the orientation of the *SHMT1* RNA GQ. The spectra of the *SHMT1* RNA GR were distinct from those of the *SHMT1* DNA GR sample, exhibiting a positive maximum at ∼265 nm and a negative minimum at ∼240 nm (Figure 5B). This signature is characteristic of a parallel GQ conformation, compared to the antiparallel and hybrid 3 + 1 structures adopted by the corresponding DNA sample. This is not unexpected, though, as RNA GQs are restricted to parallel orientation by the hydroxyl group at the 2′ carbon position.^48,49^ These results are consistent with the ^1^H NMR spectra, indicating that the RNA GQ retains the same conformation regardless of potassium concentration, unlike the DNA GQ, which shifts orientation in the presence of KCl.

Having determined that the *SHMT1* RNA GQ folds in a parallel orientation, we analyzed it using native PAGE and staining with NMM dye. The samples were prepared in the presence of increasing concentrations of KCl and compared to a GQ-positive and a GQ-negative control. When stained with NMM (Figure 5C, left), lane 7 (containing the GQ-positive control) shows a dark band, while lane 8 (GQ-negative control) is not stained, validating the specificity of NMM staining of parallel GQs. NMM staining of *SHMT1* RNA GR, lanes 1–6, revealed a lower-molecular-weight band that was visible in all samples, including when the RNA was incubated without added potassium, indicating the formation of a stable parallel GQ. The appearance of faint, higher-molecular-weight bands at KCl concentrations above 25 mM suggests the stacking of the *SHMT1* RNA GQs. When visualized after ethidium bromide staining (Figure 5C, right), all RNA bands become visible, including the GQ-negative control band in lane 8.

To investigate the stability of *SHMT1* RNA GR at each potassium concentration, we employed UV thermal denaturation spectroscopy. At each KCl concentration, a hypochromic transition corresponding to the denaturation of the *SHMT1* RNA GQ structure was observed (Figure 6A), with *T*_m_ values increasing from 51 °C at 0 mM to 77 °C at 150 mM KCl (Table S3). Assuming a two-state model, as for the *SHMT1* DNA GQ, the data was fitted to eq 1 to calculate thermodynamic parameters (Table S3). The enthalpy of formation for *SHMT1* RNA GR at 150 mM KCl was calculated to be −82.9 ± 0.1 kcal/mol. The *SHMT1* RNA GQ had higher enthalpy of formation values than the DNA GQ at all KCl concentrations. Additionally, *SHMT1* RNA GR also exhibited higher melting temperatures under all potassium-dependent conditions. These findings are consistent with reports that RNA GQs are more thermodynamically stable than DNA GQs due to their preference for parallel GQ orientation.^50^ Finally, the number of potassium ions intercalated into the *SHMT1* RNA GQ was found to be ∼3.4 (Figure 6B), from the slope of the plot of the calculated free energy of folding versus the logarithm of the potassium concentration (eq 3). This value is consistent with at least a three-planar GQ structure. Generally, potassium ions are intercalated between the G-quartets, but there are crystal structure examples of GQs where an additional potassium ion interacts with the top G-quartet, which could explain the average of >3 potassium ions we determined for the *SHMT1* RNA GQ.^51^

**Figure 6:**
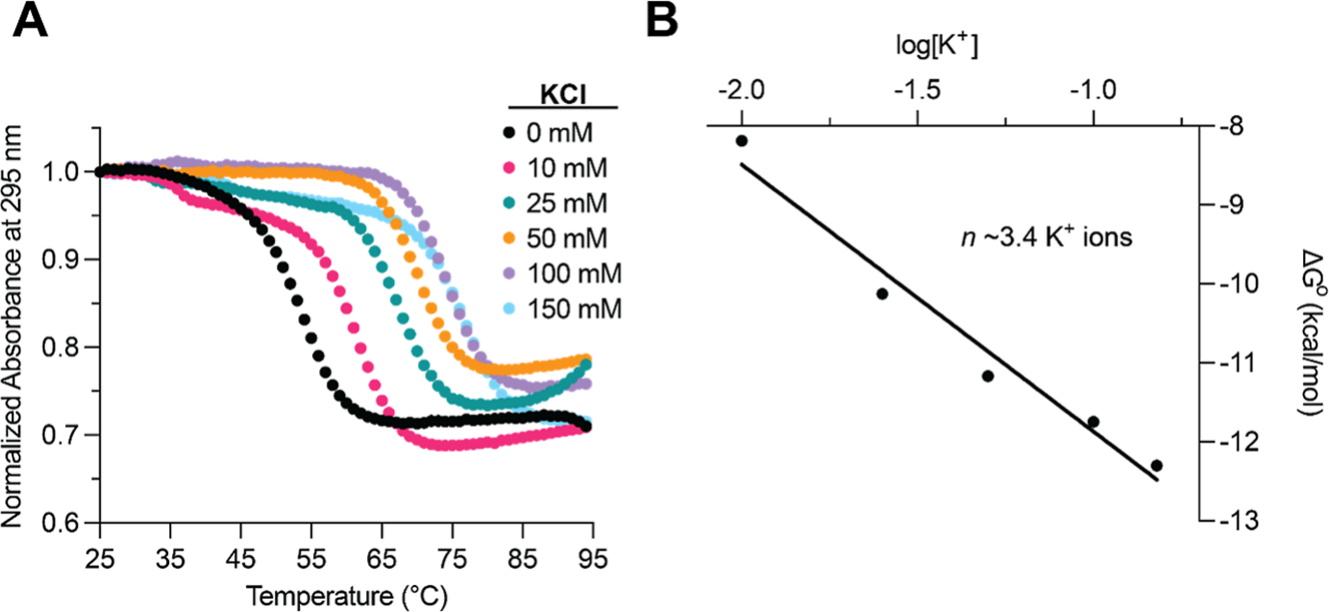
(A) KCl dependence of UV thermal denaturation results of the *SHMT1* RNA GR sequence. Hypochromic transitions of GQ melting are observed at each KCl concentration, with *T*_m_ values increasing with each KCl concentration. (B) Plot of log­[K^+^] versus Δ*G*° for *SHMT1* GR RNA UV thermal denaturation. When plotted as such, the slope of the graph is equal to the average number of potassium ions intercalated by the *SHMT1* RNA GQ.

### Transcriptional Regulation by the 5′-UTR of *SHMT1*

In summary, we showed that isolated *SHMT1* DNA/RNA GR and CR sequences form stable GQ and iM structures *in vitro*. While these experiments were in progress, a study used antibodies to map out GQ and iM structures formed in the human genome *in vivo*.^52^ Interestingly, the *SHMT1* DNA GR and CR sequences characterized here were also identified in this study for GQ and iM formation in cells, supporting the hypothesis that these structures might play a regulatory role in the expression of *SHMT1*. To test this, we assessed the impact of the full-length *SHMT1* 5′UTR on a firefly luciferase reporter gene. We constructed a plasmid that contained a *Renilla* luciferase and a firefly luciferase, where the wild-type (WT) or mutant *SHMT1* 5′UTR was cloned in front of the firefly luciferase gene. These plasmids were then transfected into A549 cells, and the relative luciferase activities were read after 72 h. We found that cells transfected with the WT *SHMT1* 5′UTR reporter had 36% less firefly luciferase activity compared to the mutant 5′UTR (Figure 7A), suggesting that the secondary structures of the *SHMT1* 5′UTR can suppress protein expression levels.

**Figure 7:**
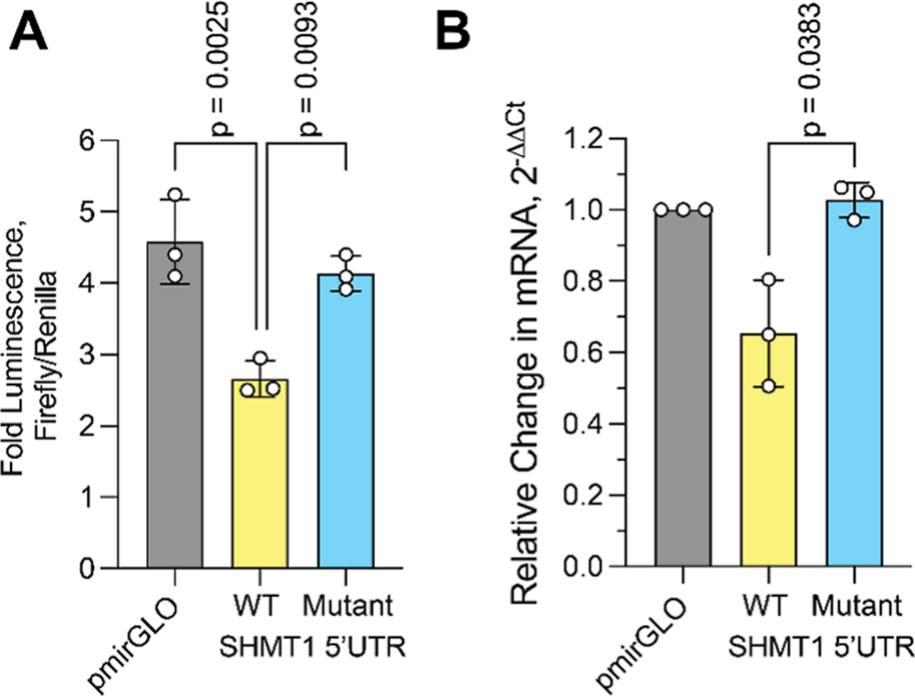
(A) Impact of *SHMT1* 5′UTR on firefly luciferase expression. A 36% reduction in relative luciferase activity in the WT indicates that DNA/RNA secondary structures play a role in suppressing gene expression. (B) *RT-qPCR* assay highlighting the suppression of firefly luciferase mRNA levels. A 37% reduction in mRNA levels in the WT suggests that DNA secondary structures play a role in regulating transcription of luciferase. All data represent the mean and standard deviation of 3 biological replicates. *P*-values are indicated for (A) one-way analysis of variance (ANOVA) with Dunnett’s correction and (B) two-tailed Welch’s *t*-test between WT and mutant.

As the mutant sequence disrupts both the GQ and iM structures simultaneously at the DNA level as well as the GQ at the RNA level, we cannot determine if the DNA GQ/iM and/or RNA GQ structures are responsible for the observed reduction in firefly activity. Therefore, we assessed the impact of the *SHMT1* DNA GR/CR sequence solely on the transcription of firefly luciferase by using *RT-qPCR* to determine relative mRNA levels as controlled by WT or mutant *SHMT1* 5′UTR sequences. Consistent with the protein activity assays, we found that cells transfected with the WT *SHMT1* 5′UTR reporter had 37% less firefly luciferase mRNA compared to the mutant 5′UTR (Figure 7B). Supporting the role of the *SHMT1* 5′UTR secondary structures in affecting luciferase transcription, the mutant sequence mRNA levels were comparable to the control pmirGLO-luciferase mRNA levels, which contained the wild-type luciferase 5′UTR (Figure 7B). Taken together, these data indicate that the GQ/iM secondary structures formed in the *SHMT1* DNA 5′UTR sequence are necessary to mediate the expression of the reporter gene by reducing transcription. Although the *SHMT1* RNA GR sequence forms a stable GQ structure *in vitro*, it is possible that the *SHMT1* 5′UTR mRNA GQ structure is not stable in the cell in the experimental conditions we used in this study, as it has been shown that stress promotes RNA GQ folding in cells, with this stress-induced folding being reversible upon stress removal.^53^ Thus, we cannot completely exclude additional regulation of *SHMT1* translation by its 5′UTR RNA GQ in conditions of cellular stress, which are well documented in MS.^54,55^

This characterization of GQ and iM structures in the *SHMT1* 5′UTR contributes to our understanding of how these secondary structures affect gene expression. While GQs and iMs are widely characterized in promoter regions,^56,57^ their regulatory roles in the 5′UTR are not as well understood. Their positioning within the 5′UTR suggests a transcriptional or translational regulatory role, but detailed mechanisms remain elusive.^56,58,59^ In our system, the presence of either, or both, secondary structures likely interferes with transcriptional machinery, resulting in lower overall mRNA levels and decreased expression of the SHMT1 protein. However, we acknowledge the limitations of our study in that our experiments were performed in a lung cancer cell line, which might not give a full picture of what happens in neurons. The regulatory effects of the GQ/iM structures are likely mediated via their interactions with proteins whose expression might differ in these two different cell types. For patients with MS, SHMT1 is overexpressed and contributes to abnormal DNA methylation. Our observation that *SHMT1* GQ/iM formation results in decreased protein expression could constitute a novel therapeutic target via stabilization of the secondary structures. Currently, there are two primary tactics used for GQ/iM targeting in cells: small molecules and nucleic acid analogue oligonucleotides.

TMPyP4 is one of the most widely used GQ-binding ligands, known to bind these structures and inhibit telomerase activity but with noticeably poor selectivity for GQs versus duplex DNA.^60^ This issue was corrected in a modified ligand, TMPyPz, which exhibited not only higher GQ selectivity but also better telomerase inhibition.^60,61^ Outside of telomere intervention, TMPyP4 and other GQ-binding ligands (pyridostatin and derivatives) were found to stabilize the formation of a GQ in the *BAZ2B* promoter in vitro.^57^ When treating the Alzheimer’s disease SH-SY5Y model cell line with these GQ ligands, Yang et al. observed a loss of BAZ2B expression that they tied directly to loss of transcription factor binding due to GQ stabilization.^57^ These small molecules are often not sequence-specific, though, with the potential to bind to a multitude of GQ-forming regions; because of this, nucleic acid analogue oligonucleotide interventions are gaining interest for the ability to design them complementary to any sequence of interest.

GQ or iM targeting oligonucleotides can be designed to stabilize or destabilize the secondary structures of interest, but because SHMT1 is upregulated in MS patients and because our results show an inhibitory effect due to GQ/iM formation, therapeutic intervention of this system would require stabilizing the *SHMT1* 5′UTR GQ/iM. One recent study designed a nucleic acid linker to stabilize the *MYC* promoter GQ, a structure that has been tied to decreased cancer progression by inhibiting protein expression.^62^ Psaras et al. designed an oligonucleotide with ends complementary to the sequences bordering the *MYC* GQ sequence, spaced by intermittent thymines to encompass the width of the GQ.^62^ This allowed the interfering DNA sequence to preferentially bind to *MYC* when the GQ is formed, stabilizing the structure in the process.^62^ Similar linkers could be designed for the *SHMT1* system, targeting the regions bordering the DNA or RNA GQ to promote structure formation. Additionally, designing a linker targeting the C-rich border sequences could promote the formation of the iM structure and result in improved downregulation of *SHMT1*. The effect of such linker oligonucleotides upon the expression of *SHMT1* will be tested in further studies in our laboratories. Thus, our study identifies novel potential therapeutic targets for the regulation of the SHMT1 expression while contributing to a growing library of 5′UTR GQ and iM structures with regulatory effects on gene expression.

## Materials and Methods

The *SHMT1* G/C-rich single-stranded DNA (ssDNA) sequences and G/C-rich mutant ssDNA sequences were chemically synthesized by Integrated DNA Technologies (IDT) (Table 1). The corresponding G-rich mRNA sequence was chemically synthesized by Horizon Discovery (Table 1). All sequences were suspended in 10 mM cacodylic acid (pH 6.5).

### Native Polyacrylamide Gel Electrophoresis (PAGE)

*SHMT1* DNA GR samples at a 20 μM concentration were prepared in the presence of varying concentrations of KCl (0, 10, 25, 50, 100, and 150 mM) in 1/2× Tris boric acid EDTA (TBE). The samples were boiled for 5 min and then cooled to 22 °C on the benchtop. A GQ-positive control sample was prepared from the brain-derived neurotropic factor (BDNF) mRNA in 150 mM KCl, and a GQ-negative control sample was prepared from the SARS-CoV-2 open reading frame (ORF) 1a RNA in 150 mM KCl (Table 1). All samples were run in a 20% native polyacrylamide gel (30:0.8 acrylamide/bis­(acrylamide)) for 4 h at 75 V and 4 °C in 1/2× TBE buffer. The gel was stained in *N*-methyl mesoporphyrin (NMM) IX, a GQ-specific dye.^43^ The gel was then stained with ethidium bromide (EtBr) to visualize all bands for comparison with the identified GQ bands.

*SHMT1* RNA GR samples (15 μM) were prepared in 1/2× TBE. A GQ from the nuclear-enriched abundant transcript 1 (NEAT1) long noncoding RNA was used as a GQ-positive control, and the SARS-CoV-2 s2m as a GQ-negative control (Table 1). The samples were prepared, run, and analyzed in the same manner as described above. All gels were performed at least in triplicate.

### Circular Dichroism Spectroscopy

To investigate the topology of the GQ/iM formation, circular dichroism (CD) spectroscopy experiments were carried out using a Jasco J-810 spectropolarimeter at 25 °C. For GQ characterization, 10 μM of *SHMT1* DNA GR was prepared in 200 μL of 10 mM cacodylic acid (pH 6.5), acquiring seven scans from 220 to 320 nm with a 1 s response time and a 2 nm bandwidth. The sample was then titrated with increasing concentrations of KCl (10, 25, 50, 100, and 150 mM KCl). The *SHMT1* DNA GR data was smoothed using the Jasco spectra analysis Savitzky–Golay filter with a convolution width of 25. *SHMT1* RNA GR was analyzed in the same manner.

For the iM characterization, the *SHMT1* DNA CR sample was prepared in 10 mM cacodylic acid, and experiments were carried out at varying pH values (4.0, 5.5, 6.5, and 7.0). Spectra were collected from 220 to 340 nm (averaging seven scans) with a 1 s response time and a 2 nm bandwidth.

### One-Dimensional Proton Nuclear Magnetic Resonance Spectroscopy

1D ^1^H NMR spectroscopy experiments were performed at 20 °C on a 500 MHz Bruker NMR spectrometer with Topspin 3.2 software (Bruker), utilizing the WATERGATE water suppression pulse sequence. 250 μM of *SHMT1* DNA GR was prepared in 10 mM cacodylic acid (pH 6.5) in a final volume of 250 μL containing 10% D_2_O. Spectra were collected after titrating with each KCl concentration (0, 10, 25, 50, 100, and 150 mM). *SHMT1* RNA GR (225 μM) was analyzed in the same manner.

The pH dependence of *SHMT1* DNA CR was carried out by preparing a 250 μM sample in 10 mM cacodylic acid and 10% D_2_O in a final volume of 250 μL. Spectra were collected as described above but at various pH values (4.0, 5.5, 6.5, and 7.0).

### Ultraviolet Thermal Denaturation Spectroscopy

UV thermal denaturation spectroscopy experiments were carried out using a Cary Series UV–vis spectrophotometer (Agilent Technologies). The stability of *SHMT1* DNA GR was evaluated as a function of various KCl concentrations by monitoring the absorbance changes at 295 nm while increasing the temperature from 25 to 95 °C at a rate of 0.2 °C per minute. *SHMT1* DNA GR was prepared at 10 μM and 200 μL in 10 mM cacodylic acid (pH 6.5), boiled for 5 min, and then cooled at 22 °C for 30 min. The protocol was repeated at each concentration of KCl (10, 25, 50, 100, and 150 mM). The same experiments were performed to evaluate *SHMT1* RNA GR stability as a function of increasing KCl concentrations.

*SHMT1* DNA CR was prepared at 10 μM and 200 μL in 10 mM cacodylic acid, boiled, and cooled at 22 °C for 30 min. The thermal denaturation experiments for this sample were performed at different pH values (4.0, 5.5, 6.5, and 7.0).

Thermodynamic parameters were obtained for GQ structures by fitting each UV thermal denaturation curve to eq 1, assuming a two-state model:^63^

1 $$A\left(T\right)=\frac{A_{U}+A_{F}\;e^{-\Delta H^{{}^{\circ}}/RT}\;e^{\Delta S^{{}^{\circ}}/R}}{e^{-\Delta H^{{}^{\circ}}/RT}\;e^{\Delta S^{{}^{\circ}}/RT}+1}$$

where *A*_U_ and *A*_F_ are the absorbances of the unfolded and native GQ, respectively, and *R* is the universal gas constant. Melting temperatures (*T*_m_) were calculated using eq 2:

2 $$T_{m}=\frac{\Delta H^{{}^{\circ}}}{\Delta S^{{}^{\circ}}}$$

The reported errors for the *T*_m_ and thermodynamic parameters were calculated based on the errors from the fit of the thermal denaturation curves to eq 1.

The number of K^+^ ions bound exclusively by the GQ structure was calculated by assuming a folded-to-unfolded GQ model in which *n* K^+^ ions are released due to heat treatment and GQ unfolding. *n* is found as the slope of a plot of Δ*G*° as a function of the logarithm of K^+^ concentration (eq 3):

$$n=\frac{dln\;K_{eq}}{dln\left[K^{+}\right]}=\frac{\Delta \Delta G^{{}^{\circ}}}{2.3RT\Delta \;\log\left[K^{+}\right]}$$ 3

where ln *K*_eq_ = (Δ*G*/*RT*) and ΔΔ*G*/Δ log­[K^+^] refers to the slope of the plot of Δ*G*° as a function of the logarithm of K^+^ concentration.^45^

### Cell Culture

All cultures of A549 cells (gift from John Minna, University of Texas Southwestern Medical School) were grown in monolayer at 37 °C/5% carbon dioxide in the DMEM (Gibco) supplemented with 10% fetal bovine serum (FBS; Corning Incorporated) and 100 units/mL penicillin and 100 μg/mL streptomycin sulfate (MP Biomedicals).

### Molecular Cloning

To clone the 5′UTR of *SHMT1* into pmirGLO (Promega), pmirGLO was digested with *Hin*dIII. The firefly luciferase gene was amplified using primers fLuc_F and fLuc_R (Table 2) to introduce an *Eco*RI recognition site after the *Hin*dIII site. The SV40 poly­(A) signal and promoter were amplified using primers SV40_F and SV40_R (Table 2) such that *Hin*dIII was removed. These fragments were ligated into pmirGLO using HiFi assembly (New England Biolabs) to generate pmirGLO-5UTR. To generate the 5′UTR of *SHMT1* (ENST00000316694.8), total RNA was isolated from A549 cells and used to generate complementary DNA (cDNA) by reverse transcription using the gene-specific primer SHMT1_R (Table 2). Following amplification with primers SHMT1_F and SHMT1_R (Table 2), the wild-type 5′UTR was ligated into pmirGLO-5UTR by HiFi assembly to generate pmirGLO-SHMT1. To generate mutant pmirGLO-SHMT1 (pmirGLO-SHMT1_MUT), which matches the G-rich mutant sequences described in the biophysical characterization, multisite directed mutagenesis was carried out by HiFi assembly using two fragments generated using primers SHMT1_F with Mut_R and SHMT1_R with Mut_F (Table 2). All plasmid sequences were verified by whole plasmid sequencing (Eurofins Scientific).

**Table 2:** Primer Sequences Used for *SHMT1* Cloning, Luciferase Assay Preparation, and Reverse Transcription Quantitative Polymerase Chain Reactions

name	sequence
fLuc_F	CCTTTCGACCTGCAGCCCAAGCTTCATGTGATCCATGAATTCGGCAATCCGGTACTGTTG
fLuc_R	GCATGCCTGCAGGTC
SV40_F	GACCTGCAGGCATGC
SV40_R	GTTGTGTCAGAAGAATCATTTGCAAAAGCCTAGGCC
SHMT1_F	CCTTTCGACCTGCAGCCCAAGCTTAAGCCCAAGCTTGG
SHMT1_R	CAGTACCGGATTGCCGAATTCATGCACTTGTTCGAAGC
Mut_F	ACGCGTTGATTCAGCCTGTCTGACACTGGTGGCACCGG
Mut_R	GTCAGACAGCGTGAATCAACGCGTCGCGCACCGCC
fLuc1_F	GACACCGGTAAGACACTGGG
fLuc1_R	GCCTCGGGGTTGTTAACGTA
fLuc2_F	GTGCAGCGAGAATAGCTTGC
fLuc2_R	TTGCTCACGAATACGACGGT
fLuc3_F	TTCGGCAACCAGATCATCCC
fLuc3_R	AGATCAAGTAGCCCAGCGTG
rLuc1_F	GGAATGGGTAAGTCCGGCAA
rLuc1_R	CCAAGCGGTGAGGTACTTGT
rLuc2_F	CGCAACTACAACGCGTACCT
rLuc2_R	GCTCCCTCGACAATAGCGTT
rLuc3_F	CCATCGTCCATGCTGAGAGT
rLuc3_R	AGGGCGATATCCTCCTCGAT

### Dual Luciferase Assays

On day 0, A549 cells were set up at a density of 1.5 × 10^4^ cells per well of a 96-well white plate (Corning Incorporated). The next day, cells were transfected with 300 ng of pmirGLO-SHMT1 or pmirGLO-SHMT1_MUT in 50 mL of growth medium using X-tremeGENE 9 (Sigma-Aldrich) according to the manufacturer’s protocol. After 72 h, firefly and *Renilla* luciferase activities were measured on a SpectraMax ID3 instrument (Molecular Device) using a Dual Luciferase Reporter Assay (Promega). Luminescence values were normalized by dividing the firefly by *Renilla*.

### Reverse Transcription Quantitative Polymerase Chain Reaction (*RT-qPCR*)

On day 0, A549 cells were set up at a density of 4 × 10^5^ cells per well of a six-well plate (Costar). The next day, cells were transfected with 1 μg of pmirGLO-5UTR, pmirGLO-SHMT1, or pmirGLO-SHMT1_MUT in 2 mL of growth medium using X-tremeGENE 9 according to the manufacturer’s protocol. After 72 h, RNA was extracted as described previously^64^ using TRIzol (Thermo Fisher) as per the manufacturer’s instruction and then treated with DNase I (Invitrogen). To prepare cDNA, 1 μg of RNA was reverse transcribed (SuperScript IV Reverse Transcriptase; Thermo Fisher) using 1 μL of 50 mM random hexamer primers (Invitrogen). For qPCR, cDNA samples were diluted 1:5 with nuclease-free water (Qiagen), and 1 μL was added to 5 μL of SYBR Green (Invitrogen), 1 μL of 10 mM primer pairs (Table 2), and 3 μL of nuclease-free water. Following 40 cycles of amplification using a QuantStudio 3 Real-Time PCR System (Applied Biosystems), RT-qPCR data was analyzed by using the ΔΔCt method, where each RNA was normalized to pmirGLO-5UTR.

## Supplementary Material



## Notes

Single- and double-stranded structure predictions for the *SHMT1* GR_MUT sequence; native PAGE gel of the *SHMT1* DNA GR sequence stained in NMM and ethidium bromide; single- and double-stranded structure predictions for the *SHMT1* DNA CR_MUT sequence; and thermodynamic parameters calculated from UV thermal denaturation of *SHMT1* DNA GR, select *SHMT1* DNA CR, and *SHMT1* RNA GR (PDF)
