# Supplementary material for: G‑Quadruplex and i‑Motif Structures in the SHMT1 5′UTR Modulate Gene Expression
Source: ACS Omega. 2026 Mar 19;11(12):19497–508. doi: 10.1021/acsomega.5c13146 (PMC13044848; doi:10.1021/acsomega.5c13146)
Supplement: Supplementary file 1 [file ao5c13146_si_001.pdf]

G-Quadruplex and i-Motif Structures in the *SHMT1* 5'UTR Modulate Gene Expression

Rosalia M. Palumbo[1,#], Manju Kasaju[1,#], Sophia C. Hershey[1], Morgan E. McCann[1], Zoe H. Woon[1,2], David B. Heisler[1*], Mihaela-Rita Mihailescu[1*]

[1]Department of Chemistry & Biochemistry, Duquesne University, Pittsburgh PA

[2]Department of Chemistry, Bryn Mawr College, Bryn Mawr PA

[#]Authors contributed equally to the work

*To whom correspondence should be addressed: mihailescum@duq.edu; heislerd@duq.edu

Supporting Information

Content

Supplemental Figures *1*

Figure S1. 1

Figure S2. 1

Figure S3. 2

Supplemental Tables *2*

Table S1. Thermodynamic Parameters for DNA GQ 2

Table S2. Thermodynamic Parameters for DNA iM 3

Table S3. Thermodynamic Parameters for RNA GQ 3

Supplemental Figures

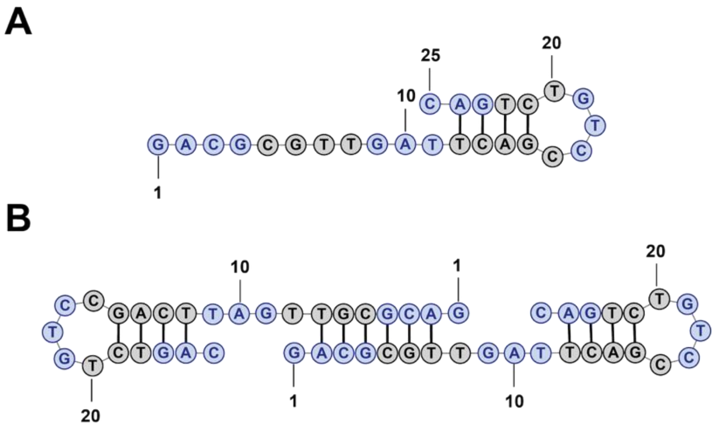

Figure S1. *(A) Single-strand RNAstructure fold of SHMT1 GR_MUT.* When folded with only one copy of the sequence, the predicted structure is an intramolecular hairpin formation*. (B) Double-strand RNAstructure fold of SHMT1 GR_ MUT.* When two copies of the sequence are included, the predicted structure is an intermolecular duplex. Mutated G-tracts predicted to form the GQ structure are colored blue, predicted base pair bonds are indicated by black connecting lines.

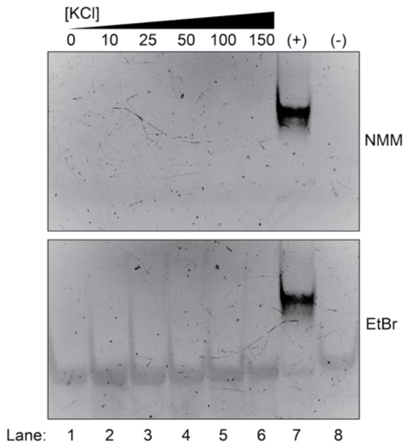

Figure S2. *(Top) SHMT1 DNA GR native PAGE with NMM staining.* The SHMT1 DNA GR was incubated with increasing concentrations of potassium but is not visible in NMM staining (lanes 1-6) compared to a GQ positive control (lane 7). *(Bottom) SHMT1 DNA GR native PAGE with EtBr staining.* Bands are visible in EtBr staining, which identifies any secondary structure formation.

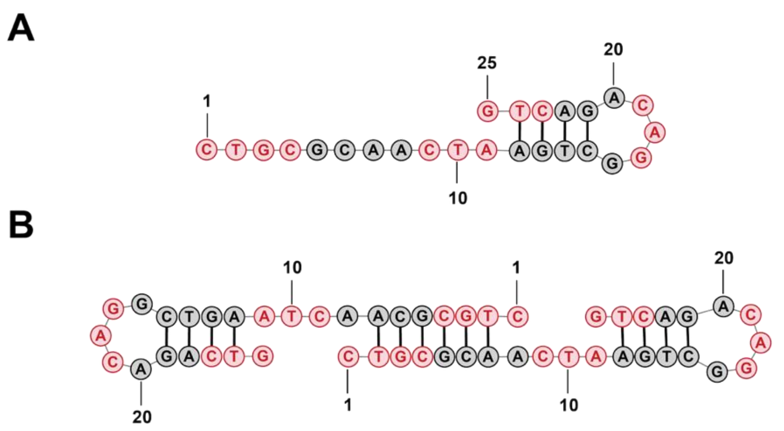

Figure S3. *(A) Single-strand RNAstructure fold of SHMT1 CR_MUT.* The predicted structure for one copy of the sequence is an intramolecular hairpin. *(B) Double-strand RNAstructure fold of SHMT1 CR_ MUT.* When folded with two copies of the sequence, the predicted structure is an intermolecular duplex. Mutated C-tracts predicted to form the iM structure are colored red, predicted base pair bonds are indicated by black connecting lines.

Supplemental Tables

Table S1. Thermodynamic Parameters for DNA GQ

KCl	T_m (°C)	ΔH°_f (kcal/mol)	ΔS°_f (cal/molK)	ΔG°_f (kcal/mol)
0 mM	34.7 ± 0.2	-33.3 ± 0.1	-108.3 ± 0.3	-1.0 ± 0.1
10 mM	56.0 ± 0.1	-61.6 ± 0.1	-187.1 ± 0.1	-5.8 ± 0.1
25 mM	58.9 ± 0.1	-66.2 ± 0.1	-199.4 ± 0.1	-6.8 ± 0.1
50 mM	62.8 ± 0.1	-64.6 ± 0.1	-192.4 ± 0.1	-7.3 ± 0.1
100 mM	68.0 ± 0.1	-66.4 ± 0.1	-194.7 ± 0.1	-8.4 ± 0.1
150 mM	71.2 ± 0.1	-64.7 ± 0.1	-188.1 ± 0.1	-8.7 ± 0.1

Table S2. Thermodynamic Parameters for DNA iM				
pH	T_m (°C)	ΔH°_f (kcal/mol)	ΔS°_f (cal/molK)	ΔG°_f (kcal/mol)
5.5	49.9 ± 0.1	-31.8 ± 0.1	-98.5 ± 0.2	-2.2 ± 0.1

Table S3. Thermodynamic Parameters for RNA GQ				
KCl	T_m (°C)	ΔH°_f (kcal/mol)	ΔS°_f (cal/molK)	ΔG°_f (kcal/mol)
0 mM	51.1 ± 0.1	-65.9 ± 0.1	-203.4 ± 0.2	-5.3 ± 0.1
10 mM	61.3 ± 0.1	-75.4 ± 0.1	-225.4 ± 0.1	-8.2 ± 0.1
25 mM	67.4 ± 0.1	-81.3 ± 0.1	-238.9 ± 0.1	-10.1 ± 0.1
50 mM	70.2 ± 0.1	-84.9 ± 0.1	-247.3 ± 0.1	-11.2 ± 0.1
100 mM	74.3 ± 0.1	-82.7 ± 0.1	-238.1 ± 0.1	-11.7 ± 0.1
150 mM	76.9 ± 0.1	-82.9 ± 0.1	-236.8 ± 0.1	-12.3 ± 0.1
